# The whole genome sequence data analyses of a *Mycobacterium tuberculosis* strain SBH321 isolated in Sabah, Malaysia, belongs to Ural family of Lineage 4

**DOI:** 10.1016/j.dib.2020.106388

**Published:** 2020-10-08

**Authors:** Jaeyres Jani, Zainal Arifin Mustapha, Chin Kai Ling, Amabel Seow Ming Hui, Roddy Teo, Kamruddin Ahmed

**Affiliations:** a Borneo Medical and Health Research Centre, Faculty of Medicine and Health Sciences, Universiti Malaysia Sabah, Sabah, Malaysia; b Department of Medical Education, Faculty of Medicine and Health Sciences, Universiti Malaysia Sabah, Sabah, Malaysia; c Department of Biomedical Sciences and Therapeutics, Faculty of Medicine and Health Sciences, Universiti Malaysia Sabah, Sabah, Malaysia; d Tuberculosis and Leprosy Control Unit, Sabah State Health Department, Kota Kinabalu, Sabah, Malaysia; e Department of Pathobiology and Medical Diagnostics, Faculty of Medicine and Health Sciences, Universiti Malaysia Sabah, Sabah, Malaysia

**Keywords:** *Mycobacterium tuberculosis*, Whole genome sequencing, Next generation sequencing, Ural family, Sabah, Malaysia

## Abstract

In 2019, 10 million new cases of tuberculosis have been reported worldwide. Our data reports genetic analyses of a *Mycobacterium tuberculosis* strain SBH321 isolated from a 31-year-old female with pulmonary tuberculosis. The genomic DNA of the strain was extracted from pure culture and subjected to sequencing using Illumina platform. *M. tuberculosis* strain SBH321 consists of 4,374,895 bp with G+C content of 65.59%. The comparative analysis by SNP-based phylogenetic analysis using maximum-likelihood method showed that our strain belonging to sublineage of the Ural family of Europe–America–Africa lineage (Lineage 4) and clustered with *M. tuberculosis* strain OFXR-4 from Taiwan. The whole genome sequence is deposited at DDBJ/ENA/GenBank under the accession WCJH00000000 (SRR10230353).

## Specifications Table

**Table:** 

Subject	Medicine and Public Health
Specific subject area	Microbiology
Type of data	Whole genome sequence with gene annotation and comparative genomic of *Mycobacterium tuberculosis* strain SBH321 Ural strain from Sabah, Malaysia.
How data were acquired	*M. tuberculosis* cultured in BACTEC MGIT system and the gDNA extracted and sequenced. *de novo* whole genome sequencing, phylogenetic and variant calling data analysis subsequently conducted.
Data format	Raw data and whole genome sequence data analysis.
Parameters of data collection	Genomic DNA from pure culture.
Description of data collection	*M. tuberculosis* identified by Genexpert MTB/RIF cultured in 7H9 Middlebrook liquid media and incubated in BACTEC MGIT system. Genomic DNA isolated using Masterpure Complete DNA and RNA purification kit. Whole genome sequencing performed by Illumina HiSeq 4000 system. Genome assembled through SPAdes version 3.11, variant calling by Genome Analysis Toolkit (GATK), and annotation by NCBI Prokaryotic Genome Annotation Pipeline (PGAP).
Data source location	Lahad Datu, Sabah, Malaysia
Data accessibility	Repository name: Mendeley data (https://data.mendeley.com/datasets/yw63xz9rgm/1) Data is publicly available at NCBI Genbank from the following links: http://www.ncbi.nlm.nih.gov/bioproject/PRJNA575111https://www.ncbi.nlm.nih.gov/biosample/SAMN12877714https://www.ncbi.nlm.nih.gov/nuccore/SRR10230353

## Value of the Data

• Since *Mycobacterium tuberculosis* of the Ural family has never been reported in Malaysia, the whole genome sequence of this strain could provide fundamental knowledge and insight towards understanding its microbial activities.
• The data could be used in the examination of the molecular characteristics and genetic variability of the *M. tuberculosis* strain which would benefit molecular epidemiologists.
• The data, an important source towards understanding the relationship between *M. tuberculosis* strains from Sabah and other regions, could assist in developing informed policy in the design and implementation of tuberculosis control programme.

## 1 Data Description

*Mycobacterium tuberculosis* is divided into seven lineages, among these, Lineage 4 is highly diversified. Several families of strains are found in this lineage and Ural amongst them [1]. The Ural family, first reported in 2005, constituted 15% of the researched strains in the Middle Ural area of Russia [2,3]. Besides Russia, these strains are mainly found in Iran, Afghanistan, Pakistan, Turkey, Kyrgyzstan, Ukraine, Abkhazia, Kazakhstan, Georgia and Armenia [1]. Apart from only one report from northern India and north eastern China, these satins have not yet been reported from any South, East, and Southeast Asia countries [1]. The Malaysian Borneo state of Sabah, located in the region of Southeast Asia where tuberculosis cases are increasing, has one of the highest cases of tuberculous in Malaysia [4]. The exact reason for this high incidence of tuberculosis is unknown; it was due to this gap of data that we undertook a project to perform whole genome sequence (WGS) analysis of *M. tuberculosis* strains from Sabah to determine their genetic characterizations.

**Fig. 1:**
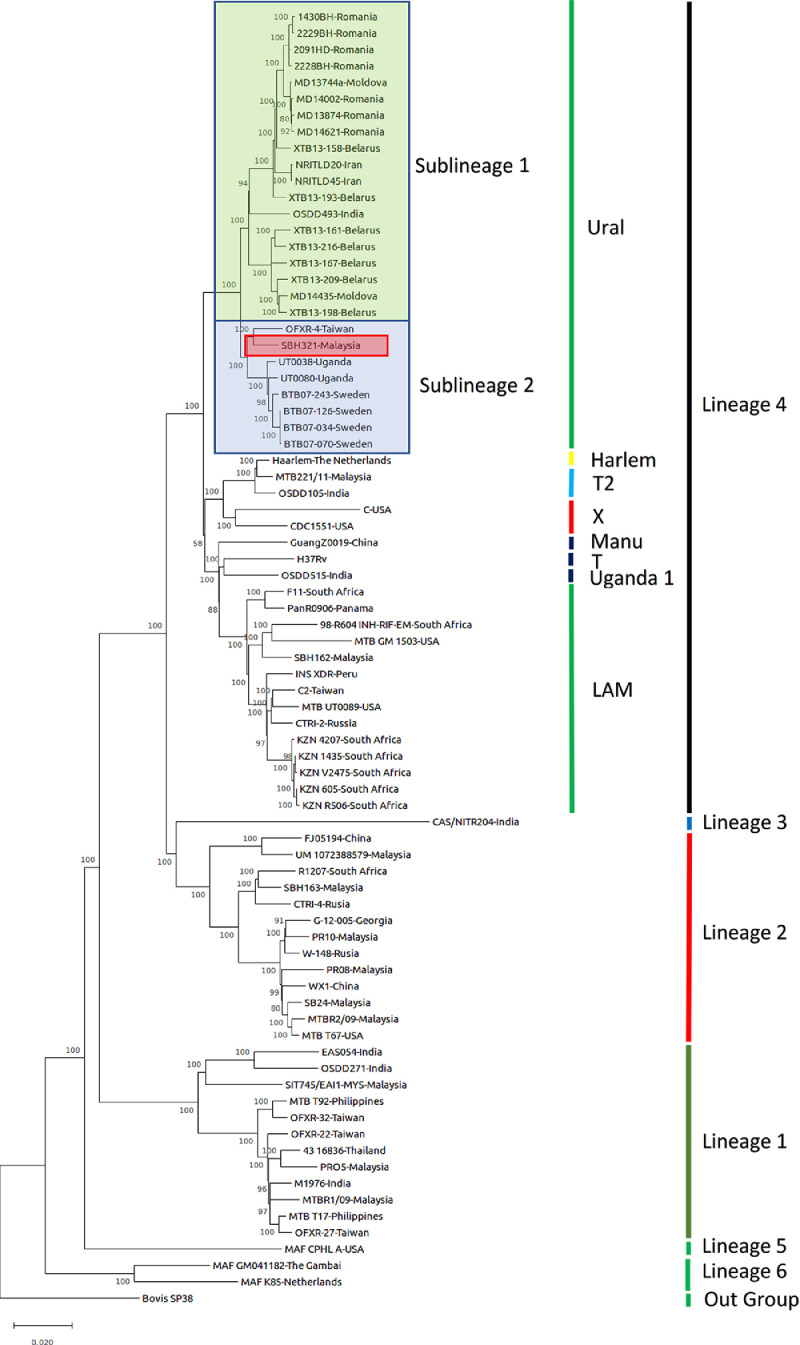
The phylogenetic tree shows that *Mycobacterium tuberculosis* strain SBH321 belongs to sublineage 2 of the Ural family in Lineage 4. The phylogenetic tree is inferred using the maximum likelihood method and General Time Reversible model by using MEGA X. The tree is rooted with Mycobacterium bovis SP38 as an outgroup.

Data analysis of the WGS of *M. tuberculosis* strain SBH321 from Sabah, Malaysia is documented in this paper. *M. tuberculosis* strain SBH321 was isolated from a 31-year-old Filipino female patient from Lahad Datu, Sabah. She was confirmed to be tuberculosis-positive by GeneXpert MTB/RIF. The strain was cultured using BACTEC MGIT system. WGS was performed by Illumina HiSeq 4000 system. The *de novo* assembly of genome generated 114 contigs with N50 of 193,257 bp. The genome size was 4,374,895 bp with 4059 predicted genes and 65.59% of G+C content. The comparative genomic analysis using the WGS of 78 strains revealed that SBH321 strain belonged to the Ural family of Lineage 4 and resembled the strains of OFXR-4 from Taiwan (Fig. 1).

## 2 Experimental Design, Materials and Methods

### 2.1 Bacterial culture and DNA extraction

The *M. tuberculosis* strain SBH321 was isolated from the sputum of a patient with tuberculosis diagnosed by GeneXpert MTB/RIF. The strain was grown in 7H9 Middlebrook medium, and incubated at 37 °C in a BACTEC MGIT 320 system (Becton-Dickinson, Oxford, United Kingdom). The genomic DNA extraction was performed using Masterpure Complete DNA and RNA purification kit (Epicenter Inc., Madison, Wisconsin, USA) according to the manufacturer's instructions but with modification in the lysis step by extending the lysis duration to 16 h. The quality of the extracted DNA was determined by Nanodrop 2000c spectrophotometer (ThermoFisher Scientific, USA).

### 2.2 Whole genome sequencing and bioinformatic analyses

99% of the genome was completely sequenced using 386 × sequencing coverage, generated a total of 11,355,058 paired reads of a 150-bp paired-end library via NEB next Ultra kit (Illumina, San Diego, CA). The sequencing data was deposited in the Sequence Read Archive (SRA) (Bio-sample accession number of SAMN12878104) and under the bio-project accession number PRJNA575111. SPAdes version 3.11.1 [5] software was used for *de novo* assembly, and NCBI Prokaryotic Genome Annotation Pipeline (PGAP) [6] software utilized to annotate the generated contigs.

### 2.3 Assembly statistic

**Table:** 

Sequencing depth	386 ×
Total length of sequences (bp)	4,374,895
Total number of contigs	114
N50 (bp)	193,257
GC (%)	65.59
CDSs	4059
tRNAs	52
5s, 16s, 23s rRNA	1, 1, 1

### 2.4 Variant calling

For the variant calling analysis, the raw sequence reads were first aligned to a reference genome, *M. tuberculosis* H37Rv (GenBank accession number NC_000962.3) using BWA MEM version 0.7.1231 [7] in SAM-BAM format. In order to convert the format into readable sequences and sort the alignments, Samtools version 0.1.1932 [8] was used. Next, Genome Analysis Toolkit (GATK) version 3.4.033 [9] performed local realignment of the sequence reads and generated the reports on variant calling analysis of the *M. tuberculosis* strain SBH321. SnpEff version 4.134 [10] was utilized for the annotation of single nucleotide polymorphism (SNP).

### 2.5 SNP-based phylogenetic genotype data of SBH321

The entire SNP matrix used in the phylogenetic analysis was performed by the Maximum Likelihood method using MEGA (Molecular Evolutionary Genetic Analysis) X [11] after aligning the nucleotide sequences using CLUSTALW [11]. The significance of the branching patterns was evaluated by bootstrap analysis of 1000 replicates. The whole genome sequence of 78 strains of *M. tuberculosis* were extracted from GenBank and were used in phylogenetic analysis [12], [13], [14] which showed that our strain belonged to the Ural family of Europe-America-Africa lineage (Lineage 4) and clustered with ofloxacin-resistant *M. tuberculosis* strain OFXR-4 from Taiwan [15].

### 2.6 Nucleotide sequence accession number

The whole genome sequence has been deposited at DDBJ/ENA/GenBank under the accession number WCJH00000000.

## Ethics Statement

This data was approved by the Ethics Committee of the Faculty of Medicine and Health Sciences, Universiti Malaysia Sabah [JKEtika 2/16 (6)].

## Declaration of Competing Interest

No competing interest.

## References

[1] Brites D., Gagneux S. (2017). The nature and evolution of genomic diversity in the Mycobacterium tuberculosis complex. Strain variation in the *Mycobacterium Tuberculosis* complex: its role in biology. Adv. Exp. Med. Biol..

[2] Mokrousov I. (2012). The quiet and controversial: Ural family of *Mycobacterium tuberculosis*. Infect. Genet. Evol..

[3] Sinkov V., Ogarkov O., Mokrousov I., Igor B., Yuri Z., Svetlana H., Scott K. (2018). New epidemic cluster of pre-extensively drug resistant isolates of *Mycobacterium tuberculosis* Ural family emerging in Eastern Europe. BMC Genom..

[4] Goroh M.M.D., Rajahram G.S., Avoi R., Boogaard C.H.A.V.D., William T., Ralph A.P., Lowbridge C. (2020). Epidemiology of tuberculosis in Sabah, Malaysia, 2012-2018. Infect. Dis. Poverty.

[5] Bankevich A., Nurk S., Antipov D., Gurevich A.A, Dvorkin M., Kulikov A.S, Lesin V.M., Nikolenko S.I, Pham S., Prjibelski A.D, Pyshkin A.V, Sirotkin A.V, Vyahhi N., Tesler G., Alekseyev M.A, Pevzner P.A (2012). SPAdes: a new genome assembly algorithm and its applications to single-cell sequencing. J. Comput. Biol..

[6] Tatusova T., DiCuccio. M., Badretdin A., Chetvernin V., Nawrocki E.P, Zaslavsky L., Lomsadze A., Pruitt K.D., Borodovsky M., Ostell J. (2016). NCBI prokaryotic genome annotation pipeline. Nucleic Acids Res..

[7] Li H., Durbin R. (2009). Fast and accurate short read alignment with Burrows-Wheeler transform. Bioinformatics.

[8] Li H., Handsaker B., Wysoker A., Fennell T., Ruan J., Homer N., Marth G., Abecasis G., Durbin R. (2009). The sequence alignment/map format and SAMtools. Bioinformatics.

[9] McKenna A., Hanna M., Banks E., Sivachenko A., Cibulskis K., Kernytsky A., Garimella K., Altshuler D., Gabriel S., Daly M., DePristo M.A. (2010). The genome analysis toolkit: a MapReduce framework for analyzing next-generation DNA sequencing data. Genome Res..

[10] Cingolani P., Platts A., Wang L.L, Coon M., Nguyen T., Wang L., Susan J.L, Lu X., Ruden D.M (2012). A program for annotating and predicting the effects of single nucleotide polymorphisms, SnpEff. Fly.

[11] Kumar S., Stecher G., Li M., Knyaz C., Tamura K. (2018). MEGA X: molecular evolutionary genetics analysis across computing platforms. Mol. Biol. Evol..

[12] Chong J., Yew S.M., Tan Y.-C., Ng K.P., Toh Y.F., Khoo J.-S., Yee W.-Y. (2015). Genome analysis of the first extensively drug-resistant (XDR) *Mycobacterium tuberculosis* in Malaysia provides insights into the genetic basis of its biology and drug resistance. PLoS One.

[13] Natalya E., Mikheecheva M.V.Z., Melerzanov A.V., Danilenko V.N. (2017). A Nonsynonymous SNP catalog of *Mycobacterium*. Genome Biol. Evol..

[14] Blouin Y., Hauck Y., Soler C., Fabre M., Vong R., Massoure P., Garnotel E. (2012). Significance of the identification in the horn of Africa of an exceptionally deep branching *Mycobacterium tuberculosis* clade. PLoS One.

[15] Zhang D., Gomez J.E, Chien J-Y., Haseley N., Desjardins C.A, Earl A.M, Hsueh P-R, Hung DT (2016). Genomic analysis of the evolution of fluoroquinolone resistance in 2 *Mycobacterium tuberculosis* prior to tuberculosis diagnosis. Antimicrob. Agents Chemother..

